# Trends in Breast Cancer Incidence and Mortality in the United States From 2004-2018: A Surveillance, Epidemiology, and End Results (SEER)-Based Study

**DOI:** 10.7759/cureus.37982

**Published:** 2023-04-22

**Authors:** Talal Bazzi, Muneer Al-husseini, Louis Saravolatz, Zyad Kafri

**Affiliations:** 1 Internal Medicine, Ascension St. John Hospital, Detroit, USA; 2 Infectious Diseases, Ascension St. John Hospital, Detroit, USA; 3 Hematology/Oncology, St. John Hospital and Medical Center, Grosse Pointe Woods, USA

**Keywords:** breast cancer incidence, breast cancer mortality, seer database, breast cancer trends, breast cancer research

## Abstract

The incidence and mortality data for patients with breast cancer in the United States are important to healthcare administrators for planning healthcare measures such as screening mammograms. In this study, we examined breast cancer incidence and incidence-based mortality in the United States from 2004-2018 using the Surveillance, Epidemiology, and End Results (SEER) database. We reviewed 915,417 cases of breast cancer diagnosed between 2004 and 2018. Overall, the data showed an increased incidence rate of breast cancer among all races and a decreased mortality rate among all races. Breast cancer incidence rates increased by 0.3% (95% CI, 0.1, 0.4, p<0.001) per year over the study period. Breast cancer incidence rates increased for all age, race, and stage groups except for stage regional, which showed a statistically significant decrease in the incidence of -0.9% (95% CI, -1.1, -0.7, p<0.001). The highest decrease in mortality was observed among white patients, with an overall statistically significant decrease in rates by -14.3% (95% CI, -18.1, -10.4, p <0.001). The highest decrease in rates was observed between 2016 and 2018: -48.6 (95% CI, -52.6, -44.3, p <0.001). In black/African American patients, the overall incidence-based mortality decreased by -11.6% (95% CI -15.9, -7.1 p <.001), with the highest decrease in rates observed between 2016 and 2018 with a decrease of -51.3% (95% CI -56.6, -45.3, p <0.001). In Hispanic Americans, the overall incidence-based mortality decreased by -12.3% (95% CI -16.9, -7.4, p <.001), which is lower than in white Americans.

## Introduction

The incidence of breast cancer has been increasing in the United States [1,2]. Each year, 264,000 new cases of breast cancer are diagnosed in women, and about 2,400 cases are diagnosed in men in the United States [3]. As it is the most common diagnosis of cancer in women in the United States, one can imagine the burden this disease carries in terms of healthcare resources being used up. Understanding why the incidence continues to increase is of the utmost importance and remains a mystery. Some theories as to why the incidence continues to increase could be a result of overdiagnosis by screening mammograms with their effectiveness in detecting a greater number of small tumors or because of a general increase in the incidence of breast cancer for other reasons [3]. This study aims to examine the trends in the incidence and incidence-based mortality rates of breast cancer in the United States using the Surveillance, Epidemiology, and End Results (SEER) database. By figuring out what the incidence of breast cancer is in this year’s interval and then determining which ethnic population is highest at risk for breast cancer, clinicians can begin to push for more cancer screening for these individuals. 

## Materials and methods

Data source

The SEER database is the largest cancer database in the US. The data were obtained from registries across the US. The data represent about 48% of the US population. Approximately more than 100 million Americans, among whom more than nine million are cancer patients. The SEER registry collected demographic and cancer diagnosis information from SEER’s 9 registry areas, including California (San Francisco and Oakland), Connecticut, Georgia (Atlanta only), Hawaii, Iowa, Michigan (Detroit only), New Mexico, Utah, and Washington (Seattle and Puget Sound region), which cover approximately 10% of the US population. Data about breast cancer deaths were derived from information recorded in death certificates and ascertained from the US Centers for Disease Control and Prevention’s (CDC) National Center for Health Statistics (NCHS). The SEER data provide information that includes stages of cancer at the time of diagnosis, survival data, and mortality data. The mortality data is provided by the National Center for Health Statistics. The data also utilizes the Census Bureau database to calculate the rate of cancer among the US population. The National Cancer Institute (NCI) and the U.S. Centers for Disease Control and Prevention (CDC) are the funding institutions for the program. We used SEER*Stat software (version 8.3.9) to analyze breast cancer cases diagnosed during 2004-2018 from the SEER 18 registries [4].

Study population

Patients diagnosed with breast cancer between 2004 and 2018 will be the study population of this research article. For this selection, we used the ‘Site Recode ICD-O-3/WHO 2008 classification’ and ‘Histology Recodes - Broad Groupings’ variables. Ductal carcinoma in situ (DCIS) was identified using ICD-O-3 codes (8201/2, 8230/2, 8401/2, 8500/2, 8501/2, 8503/2, 8504/2, 8507/2, 8521/2, 8522/2, and 8523/2). Patients whose diagnoses relied on an autopsy or death certificate were excluded from the study. In this study, we investigated the following variables: age at diagnosis, race, state (where the patient lives), stage at diagnosis (using SEER historic stage A), and site of the tumor within the breast (using the “primary site” variable).

Outcomes

Two main outcomes were calculated in this study: incidence and incidence-based mortality rates. All rates were adjusted to the 2000 US standard population and expressed in 100,000 person-years. These rates were calculated during 2000-2018 according to demographic and tumor characteristics. Incidence-based mortality rates were calculated as the number of breast cancer deaths among cases diagnosed over time among people at risk in the SEER areas [5]. 

Then we calculated the annual percentage changes (APCs) of incidence and incidence-based mortality rates over the study period according to baseline demographic and tumor characteristics.

Statistical analysis

The SEER*Stat software (version 8.3.9) was used to calculate all incidence and incidence-based mortality rates. The National Cancer Institute’s Joinpoint Regression program, version 4.5.0.1, was used to calculate APCs [6]. The Joinpoint Regression software uses t-tests to determine if APCs are statistically significant from zero; a p-value less than 0.05 was considered to indicate statistical significance. This analysis program selected the best-fitting log-linear regression model to identify the conjunctures (calendar years) when annual percentage changes (APC) changed significantly, allowing for the minimum number of joinpoints necessary to fit the data. APC was calculated as (exp[β]-1)*100, where the regression coefficient (β) was estimated by fitting a least-squares regression line to the natural logarithm of the rates, using the calendar year as a regressor variable. The software then analyzes rates over time and detects significant changes in APCs, then selects the best model with the minimum number of joinpoints [7]. All statistical tests were two-sided. IRB approval was obtained from the Ascension St. John Institutional Review Board.

## Results

Baseline characteristics

We reviewed 915,417 cases diagnosed from 2004-2018 (Table 1). Most of these cases were among white patients (634,968 [69.36%]), patients older than 40 years (871064 [95.15%], and patients with a localized stage (581532 [63.53%]) (Table 1).

**Table 1:** Breast Cancer incidence and mortality rates in the US (2004-2018) A) Cases included first primary tumors that matched the selection criteria, were microscopically confirmed, and were not identified only from autopsy records or death certificates. B) using SEER Historic Stage A

Characteristic	Incidence of Breast Cancer		Mortality of Breast Cancer	
	Cases, No (%)^a^	Rate	Cases, No (%)^a^	Rate
Overall	915417 (100)	176	215679 (100)	39.2
Age at diagnosis, y				
< 40	44353 (4.85)	27.1	4275 (1.98)	2.6
>40	871064 (95.15)	273.8	211404 (98.01)	63.2
Race				
White	634968 (69.36)	188.3	154774 (71.76)	40
Black	98495 (10.76)	179	29716 (13.78)	56.8
Asian or Pacific Islander	73943 (8.08)	142.7	11162 (5.18)	22.1
American Indian American/Alaska Native	4921 (0.54)	131.9	1188 (0.55)	35.8
Hispanic	98342 (10.74)	133.3	18617 (8.63)	29.1
Stage at diagnosis^b^				
Localized	581532 (63.53)	111.2	97811 (45.35)	17.4
Regional	264687 (28.91)	51.7	72259 (33.50)	13.3
Distant	52135 (5.70)	9.9	36778 (17.05)	6.9

During the study period, 215,679 women died of breast cancer and were included in the incidence-based mortality analysis (Table 1). Most of these deaths occurred in cases older than 40 years (211,404 [98.01%]), white patients (154774 [71.76%]), and patients who had a localized stage (97811 [45.35%]) (Table 1).

Incidence rates and trends over time

Breast cancer incidence rates were highest among the age group >40 years (273.8), white patients (188.3), and localized stages (111.2) (Table 1).

Breast cancer incidence rates increased by 0.3% (95% CI, 0.1, 0.4, p<0.001) per year over the study period. Breast cancer incidence rates increased for all age, race, and stage groups except for stage regional, which showed a statistically significant decrease in incidence by -0.9% (95% CI, -1.1, -0.7, p<0.001).

Rates for white patients increased by 0.2% (95% CI, 0.1, 0.4, p<0.001), and a similar trend was observed in black/African American patients by 0.5% (95% CI, 0.2, 0.8, p<0.001), Asian or Pacific Islanders by 1.2% (95% CI, 1.0, 1.5, p<0.001) and Hispanics by 0.7% (95% CI 0.4, 1.0, p<0.001). In American Indian/Alaska Native patients, there was a non-significant decrease in incidence between 2004-2006 and 2012-2018; however, a significant increase in incidence was observed between 2006-2012 at 4.2% (95% CI, 1.5, 6.9, p<0.001).

Incidence-based mortality rates and trends over times

Breast cancer incidence-based mortality rates were highest among the age group >40 years (63.2), black/African American patients (56.8), and localized stage (17.4) (Table 1).

Breast cancer incidence-based mortality decreased by -13.7% (95% CI, -17.7, -9.5, p <0.001) per year over the study period.

As shown in Table 2, breast cancer incidence-based mortality showed a statistically significant decrease by -4.7% (95% CI, -7.5, -1.8, p <0.001) in the age group <40 years and decreased by -13.7% (95% CI, -17.5, -9.7, p <0.001) in the age group >40 years. The highest statistically significant decrease was observed between 2016 and 2018 among the age group >40 years, with a statistically significant decrease of -48.3% (95% CI, -52.4, -44.0, p <0.001).

**Table 2:** Trends in breast cancer incidence-based mortality rates in the US (2004-2018) A) Annual percentage changes, calculated using Joinpoint regression software. B) A two-sided p-value was calculated using the t-test to determine the significance of the APC change. C) using SEER Historic Stage A

	Overall (1992-2015)		Trends								
			1			2			3			
	APC^a^ (95% CI)	P value^b^	year	APC^a^ (95% CI)	P value^b^	year	APC^a^ (95% CI)	P value^b^	year	APC^a^ (95% CI)	P value^b^
Overall											
Age at diagnosis, y											
<40	-4.7 (-7.5 - -1.8)	<0.001	2004-2015	-2 (-4.3 - 0.4)	0.1	2015-2017	-30.5 (-51.3 - -0.6)	<0.001			
>40	-13.7 (-17.5 - -9.7)	<0.001	2004-2012	-5.9 (-6.7 - -5)	<0.001	2012-2016	-17.6 (-20.9 - -14.2)	<0.001	2016-2018	-48.3 (-52.4 - -44)	<0.001
Stage at diagnosis^c^											
Localized	-16.8 (-20.8 - -12.6)	<0.001	2004-2012	-8.2 (-9.5 - -6.8)	<0.001	2012-2016	-21.7 (-26.7 - -16.5)	<0.001	2016-2018	-51.9 (-57.8 - -45.2)	<0.001
Regional	-12 (-14.9 - -9)	<0.001	2004-2011	-6 (-7.4 - -4.5)	<0.001	2011-2015	-14.5 (-19.3 - -9.3)	<0.001	2015-2017	-36 (-43 - -28.1)	<0.001
Distant	-4 (-6.6 - -1.4)	<0.001	2004-2011	1.7 (-0.4 – 3.8)	0.1	2011-2016	-5.6 (-10.1 - -0.9)	<0.001	2016-2018	-29.3 (-39.4 - -17.5)	<0.001
Race											
White	-14.3 (-18.1 - -10.4)	<0.001	2004-2012	-6.5 (-7.3 - -5.7)	<0.001	2012-2016	-18.4 (-18.4 - -21.6)	<0.001	2016- 2018	-48.6 (-52.6 - -44.3)	<0.001
Black	-11.6 (-15.9 - -7.1)	<0.001	2004-2013	-4.1 (-5.1 - -3.1)	<0.001	2013-2016	-17 (-26 - -6.8)	<0.001	2016-2018	-51.3 (-56.6 - -45.3)	<0.001
Asian or Pacific Islander	-11.7 (-15.9 - -7.4)	<0.001	2004-2015	-5.4 (-7.2 - -3.6)	<0.001	2015-2018	-43.2 (-50.5 - -34.7)	<0.001			
Indian American/Alaska Native	-10.9 (-14.8 - -6.8)	<0.001	2004-2012	-2.6 (-5.6 - -0.4)	0.1	2012-2016	-16 (-27 - -3.4)	<0.001	2016-2018	-44.6 -58.2 - -26.7)	<0.001
Hispanic	-12.3 (-16.9 - -7.4)	<0.001	2004-2016	-6.9 (-8.5 - -5.3)	<0.001	2016-2018	-59.3 (-69.5 - -45.8)	<0.001			

The highest decrease in mortality was observed among white patients, with an overall statistically significant decrease in rates by -14.3% (95% CI, -18.1, -10.4, p <0.001). The highest decrease in rates was observed between 2016 and 2018 by -48.6 (95% CI, -52.6, -44.3, p <0.001). In black/African American patients, the overall incidence-based mortality decreased by -11.6% (95% CI -15.9, -7.1 p <.001), with the highest decrease in rates observed between 2016 and 2018 with a decrease of -51.3% (95% CI -56.6, -45.3, p <0.001). In Hispanic Americans, the overall incidence-based mortality decreased by -12.3% (95% CI -16.9, -7.4 p <.001), which is lower than in white Americans.

## Discussion

In the study, we reviewed 915,417 cases of breast cancer diagnosed between 2004 and 2018, as shown in Table 1. Most of these cases were found in white females, at roughly 70%. Approximately 95% of these patients were older than 40 years, and roughly 64% of them had localized breast cancer. In terms of incidence, black/African American patients had an incidence of 10.8%, Hispanics had an incidence of 10.7%, and Asian or Pacific Islanders had an incidence of 8.1%. Overall, breast cancer incidence rates rose roughly by 0.3% per year over the study period. Breast cancer incidence rates increased for all ages, races, and stages except among stage regional patients who had breast cancer rates, which showed a statistically significant decrease in the incidence of 0.9% over the study period. Among races, the incidence rate for white patients increased by 0.2%, 0.5% for black/African-American patients, 1.2% for Asian or Pacific Islanders, and 0.7% for Hispanic patients. In American Indian/Alaska Native patients, there was a non-significant decrease in incidence between 2004-2006 and 2012-2018; however, a significant increase in incidence was observed between 2006 and 2012 at 4.2%.

Several risk factors are believed to contribute to the increasing incidence of breast cancer in the United States. Obesity is one of the main risk factors associated both with a higher risk of developing breast cancer, particularly in postmenopausal women, and with worse disease outcomes for women of all ages. The million-woman study looked at 1.2 million women from the United Kingdom (UK), aged 50 to 64 years, with a mean of 5.4 years, including 45,037 women with breast cancer, finding a nearly 30% higher risk of developing postmenopausal breast cancer with obesity [8]. Similarly, a meta-analysis of 34 studies comprising more than 2.5 million women, including roughly 24,000 postmenopausal breast cancer women, indicated that postmenopausal breast cancer risk was positively associated with each 5 kg/m² increase in body mass index (BMI) [9,10].

Another potential risk factor is the decrease in fertility rates. The average age at which a woman would have her first child in the US in the 1960s was roughly 23 years. More recently, the average age at which a woman will have her first child is 27 years old [11]. Millennial women are delaying having children for several reasons, which include a focus on careers and financial stability. The delay in having children is leading to higher estrogen exposure over the women’s lifetime and therefore increases the chance of such women developing breast cancer.

In terms of incidence-based mortality, the highest decrease in mortality was observed among the white population overall as compared to other populations such as blacks/African Americans, or Hispanics. This could be secondary to the lack of medical coverage, barriers to early detection and screening, more advanced stages of disease at diagnosis among minorities, and unequal access to improvements in cancer treatment [12]. 

It is known that low socioeconomic status is associated with worse medical outcomes, specifically cancer screening and primary prevention, as compared to other patients who are more affluent. Black/African American, and Hispanic patients may be affected by this to a greater degree than white patients. In 2020, nearly 19.2% of black/African Americans and 14% of Hispanics lived in poverty, compared to 8.2% of white Americans [13]. 

A study was done in 2014, The Carolina Breast Cancer Study, found a significant racial and ethnic difference in physical activity among breast cancer survivors, showing that black/African American women and Hispanic women, compared to white women, are much less likely to meet national physical activity guidelines after diagnosis [14]. This could be another reason why mortality rates are higher among minorities as compared to whites. Another study done by the Northeast Ohio Breast Cancer Survivors Foundation found roughly a 13% decrease in physical activity in African Americans as compared to white individuals after high school completion. Patients diagnosed with breast cancer, no matter their race or ethnicity, should be educated by their healthcare provider about the importance of exercise at the time of diagnosis.

These findings can be used to aid healthcare policy, cancer surveillance, and cancer disparity. Given these findings, clinicians can begin to target specific individuals in certain populations to help catch breast cancer sooner. By catching cancer sooner, clinicians can increase the chance of overall survival. This study also raises some questions about cancer disparities.

There were some limitations to this study. Using the SEER database, there are some underreported and incomplete data in variables that are not recorded. Some patients register in and out of the SEER database, and this leads to variations in the reporting of data. This is an unfortunate aspect of the database. There are also a lot of positive aspects to using SEER. It is the most comprehensive cancer surveillance system in the US. All these strengths are available on the CDC SEER website. Large sample size: SEER covers approximately 34.6% of the US population and includes data on more than 35% of all incident cancer cases in the US, making it a valuable resource for studying rare cancers and analyzing population-level trends. Longitudinal data: SEER has been collecting cancer data since 1973, providing a wealth of longitudinal data on cancer incidence and trends over time. Comprehensive data collection: SEER collects detailed data on patient demographics, tumor characteristics, treatment, and outcomes, allowing for comprehensive analyses of breast cancer incidence and survival. High-quality data: SEER data undergoes rigorous quality control procedures, including data verification, coding, and auditing, ensuring that the data is reliable and accurate. Standardized coding: SEER uses standardized coding systems, such as the International Classification of Diseases for Oncology (ICD-O), ensuring consistency in data collection and making it easier to compare data across different regions and periods.

## Conclusions

Overall, the data showed an increased rate of breast cancer detection among all races and ethnicities and a decreased mortality rate among all races and ethnicities. Black/African American patients and Hispanic patients had the lowest rate of decrease in terms of mortality as compared to white patients and the highest rise in the incidence of newly diagnosed breast cancer as compared to white patients. This could be explained by differences in socioeconomic status, having more barriers to receiving primary prevention care and having overall more aggressive forms of cancer at the time of diagnosis. These findings help reduce cancer disparities and improve public health as they give clinicians insights into which population group is most at risk of developing breast cancer. Providers can also begin to increase cancer screening via mammograms in patient populations that are at increased risk. This can help in the battle of eliminating cancer in the future. 
